# Estimating the Threshold Surface Density of Gp120-CCR5 Complexes Necessary for HIV-1 Envelope-Mediated Cell-Cell Fusion

**DOI:** 10.1371/journal.pone.0019941

**Published:** 2011-05-27

**Authors:** Shiva Naresh Mulampaka, Narendra M. Dixit

**Affiliations:** 1 Department of Chemical Engineering, Indian Institute of Science, Bangalore, India; 2 Bioinformatics Centre, Indian Institute of Science, Bangalore, India; Dana-Farber Cancer Institute, United States of America

## Abstract

Reduced expression of CCR5 on target CD4^+^ cells lowers their susceptibility to infection by R5-tropic HIV-1, potentially preventing transmission of infection and delaying disease progression. Binding of the HIV-1 envelope (Env) protein gp120 with CCR5 is essential for the entry of R5 viruses into target cells. The threshold surface density of gp120-CCR5 complexes that enables HIV-1 entry remains poorly estimated. We constructed a mathematical model that mimics Env-mediated cell-cell fusion assays, where target CD4^+^CCR5^+^ cells are exposed to effector cells expressing Env in the presence of a coreceptor antagonist and the fraction of target cells fused with effector cells is measured. Our model employs a reaction network-based approach to describe protein interactions that precede viral entry coupled with the ternary complex model to quantify the allosteric interactions of the coreceptor antagonist and predicts the fraction of target cells fused. By fitting model predictions to published data of cell-cell fusion in the presence of the CCR5 antagonist vicriviroc, we estimated the threshold surface density of gp120-CCR5 complexes for cell-cell fusion as ∼20 *µm* ^−2^. Model predictions with this threshold captured data from independent cell-cell fusion assays in the presence of vicriviroc and rapamycin, a drug that modulates CCR5 expression, as well as assays in the presence of maraviroc, another CCR5 antagonist, using sixteen different Env clones derived from transmitted or early founder viruses. Our estimate of the threshold surface density of gp120-CCR5 complexes necessary for HIV-1 entry thus appears robust and may have implications for optimizing treatment with coreceptor antagonists, understanding the non-pathogenic infection of non-human primates, and designing vaccines that suppress the availability of target CD4^+^CCR5^+^ cells.

## Introduction

The entry of HIV-1 into target cells requires the formation of complexes between the viral envelope protein (Env) and the cellular receptor, CD4, as well as a coreceptor, either CCR5 or CXCR4. CCR5 appears to play a central role in HIV-1 transmission and disease progression to AIDS. Viruses transmitted across individuals are predominantly R5 viruses, i.e., require CCR5 for entry [1], [2]. Studies of simian immunodeficiency virus (SIV) infections of non-human primates suggest that differences in the expression level of CCR5 on target CD4^+^ cells may underlie the difference between the non-pathogenic infection of natural hosts, such as African green monkeys and sooty mangabeys, and the pathogenic infection of non-natural hosts, such as rhesus macaques [1]–[3]. The former have substantially lower levels of CD4^+^CCR5^+^ target cells than the latter [4]. Low CCR5 expression may imply reduced susceptibility of cells to infection [5], [6]. Consequently, the extent of viral replication at mucosal sites may be suppressed, lowering the probability of transmission. Indeed, low CCR5 expression in newborns correlated with poor SIV transmission via breast-feeding, which may underlie the negligible mother-to-child transmission of infection in natural hosts [7]. Similarly, humans homozygous for the CCR5Δ32 allele, which results in complete suppression of CCR5 expression, are extraordinarily resistant to HIV-1 infection [8]. At the same time, low CCR5 expression may control damage to the gut mucosa, suppressing microbial translocation, and also reduce T cell homing to sites of inflammation, thereby lowering immune activation and contributing to the non-pathogenic nature of infection in natural hosts [4], [9], [10]. Reducing the availability of target CD4^+^CCR5^+^ cells therefore appears to be a promising strategy for therapeutic and preventive vaccine development [1]–[3]. Indeed, the CCR5 antagonist maraviroc was found recently to protect rhesus macaques from vaginal transmission (Veazey et al., Abstract # 84LB 17th Conference on Retroviruses and Opportunistic Infections, 2010).

Env is a trimer of non-covalently attached extracellular gp120 and transmembrane gp41 glycoprotein heterodimers [11]. During viral entry, gp120 first binds to CD4, following which conformational changes expose a cryptic binding site on gp120 for CCR5 [12]. Following CCR5 binding to gp120, further conformational changes bring the viral and cell membranes into close apposition, culminating in viral entry [12]–[14]. Direct observation of the protein complexes that mediate viral entry has remained a challenge. One strategy to overcome this limitation has been to employ mathematical models to analyse viral infectivity assays and infer the stoichiometry and/or the number of complexes necessary for viral entry [14]–[21]. Following such an approach, previous studies have argued that multiple CD4 and CCR5 molecules must be bound to gp120 for viral entry [15], [16]. More recent studies using virions expressing heterotrimeric Env containing combinations of wild-type and mutant gp120 molecules, the latter incapable of mediating entry, suggested that a single Env trimer with at least two functional gp120 subunits is adequate for HIV-1 entry [17], [18]. When the latter experiments were reanalysed using more detailed mathematical models, one study estimated that 5 trimers on a virion carrying 9 trimers are necessary for entry [19], whereas another study estimated that 8 trimers (range 2–19 depending on the assumptions employed) represent the threshold for entry [20]. Further, a model of allosteric interactions between CCR5 and gp120 argued that the better adapted a viral strain is to utilize CCR5, the fewer the CCR5 molecules needed for entry, with highly adapted strains requiring a single CCR5 bound to gp120 [14]. Robust estimates of the threshold number and the stoichiometry of Env-CD4-CCR5 complexes necessary for HIV-1 entry are thus lacking.

Here, we developed a mathematical model that mimics cell-cell fusion assays widely employed to investigate HIV entry into target cells (e.g., [13], [22]–[25]). The model employs a reaction network-based approach to describe the protein interactions that precede viral entry and quantitatively predicts the influence of the CCR5 expression level on the susceptibility of target cells to Env-mediated cell-cell fusion. We applied the model to analyse data from cell-cell fusion assays in the presence of the CCR5 antagonist vicriviroc and obtained estimates of the threshold surface density of gp120-CCR5 complexes necessary for cell-cell fusion. We validated the estimate by comparison of model predictions with independent data from cell-cell fusion assays in the presence of rapamycin, which down-regulates CCR5 expression, as well as assays using different Env clones in the presence of another coreceptor antagonist, maraviroc.

## Results

### Model formulation

**Figure 1:**
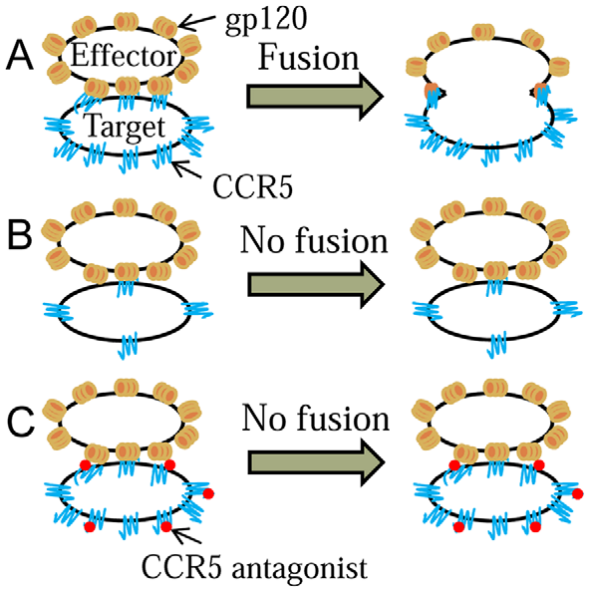
Schematic of the model. A) High CCR5 expression on a target cell allows the formation of the requisite gp120-CCR5 complexes for cell-cell fusion. B) Low CCR5 expression or C) the presence of a coreceptor antagonist reduces the surface density of gp120-CCR5 complexes and prevents fusion.

We modelled cell-cell fusion assays where target cells expressing CD4 and CCR5 are exposed to effector cells expressing Env in the presence of a coreceptor antagonist and the percentage of target cells fused with effector cells is measured (e.g., see [23], [24]). To describe these assays, we first considered a single target cell-effector cell pair in close apposition and employed reaction kinetics to determine the surface densities of different Env-CD4-CCR5 complexes formed across the pair. The reaction network and the rate equations are mentioned in Text S1. We found by solving the rate equations that reaction equilibrium was attained rapidly (∼1 s) compared to the time required for cell-cell fusion (∼min) (Fig. S1). Further, for typical CD4 and Env expression levels, CD4 appeared to be in large excess so that all gp120 monomers were bound to CD4 at equilibrium. The reaction network may therefore be simplified by ignoring the trimeric nature of Env and considering the total surface density of gp120 molecules as being available for interaction with CCR5. With this simplification, we determined the surface density of gp120-CCR5 complexes formed across a closely apposed target cell-effector cell pair as a function of the CCR5 expression level on the target cell (Methods). We postulated that a threshold surface density of gp120-CCR5 complexes must be formed for cell-cell fusion. Thus, if the surface density of gp120-CCR5 complexes formed is larger than the threshold, the target cell-effector cell pair is fused (Fig. 1). We next assumed that the CCR5 expression level on target cells in a cell-cell fusion assay follows a truncated normal distribution. Cells with smaller expression levels of CCR5 form fewer complexes and may not fuse. We thus estimated the fraction of cells that expresses CCR5 at levels larger than that required to form the threshold surface density of gp120-CCR5 complexes, which yields the fraction of cells fused in the assay.

A coreceptor antagonist typically binds to an allosteric site on CCR5 and inhibits CCR5 binding to gp120 [26]. Consequently, fewer gp120-CCR5 complexes are formed between a cell-cell pair as exposure to the coreceptor antagonist increases (Fig. 1). A target cell would therefore require higher CCR5 expression to form the threshold surface density of gp120-CCR5 complexes when exposed to the coreceptor antagonist. Thus, in a cell-cell fusion assay, the fraction of cells fused decreases as the concentration of the coreceptor antagonist increases. We employed the standard ternary complex model to describe the gp120-CCR5 interaction across a cell-cell pair in the presence of a coreceptor antagonist [27]. Accordingly, we estimated the fraction of cells fused at different levels of exposure to the antagonist (Methods). We present model predictions below.

### Model predictions

#### Distribution of CCR5 on target cells

**Figure 2:**
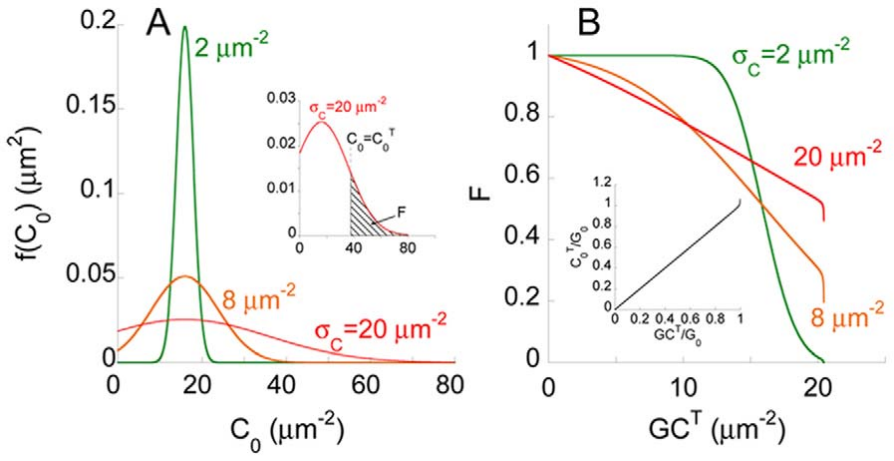
Model predictions of cell-cell fusion in the absence of a coreceptor antagonist. A) Distribution, *f*(*C*_0_), of the CCR5 expression level, *C*_0_, across cells, predicted using Eq. (12), for the mean expression, 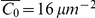 and different values of the standard deviation, 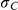. Inset: Fraction of cells fused, *F*, is the (shaded) area under the *f*(*C*_0_) curve for 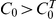, the threshold CCR5 expression level for fusion. B) *F* as a function of *GC^T^*, the threshold surface density of gp120-CCR5 complexes necessary for fusion. Inset: The dependence of 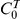 on *GC^T^* computed using Eq. (11).

In Fig. 2A, we present the distribution, *f*(*C*_0_), of the CCR5 expression level, *C*_0_, on cells, computed using Eq. (12) (Methods), for a fixed mean expression level 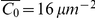, which corresponds to ∼5000 molecules/cell (radius∼5 *µm*), and different standard deviations, 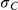. For small values of 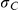 (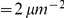 in Fig. 2A), the distribution is nearly normal and sharply peaked at 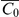. As 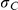 increases, the size of the peak drops and the distribution spreads over a broader range of values of 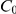 centred near 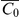. For even larger values of 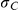 (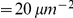 in Fig. 2A), because the distribution is truncated at 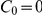, the distribution spreads to larger values of 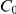 and is unevenly distributed about 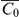.

#### Threshold CCR5 expression and cell-cell fusion

With the above distribution of CCR5 expression and given a threshold CCR5 expression level necessary for fusion, 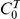, we computed the fraction of cells fused in a cell-cell fusion assay, 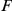, using Eq. (13) (area of the shaded region in Fig. 2A (inset)). 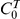 depends on the threshold surface density of gp120-CCR5 complexes that enables entry, 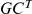 (Eq. (11)). We therefore examined model predictions of the dependence of 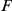 on 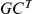 for different values of 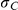 (Fig. 2B). 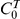 increases upon increasing 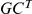 (Fig. 2B (inset)). Thus, for a fixed 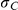, increasing 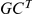 resulted in smaller *F* (Fig. 2B). When 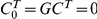, all cells had 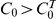, which implied *F* = 1. As 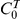 increased, a smaller fraction of cells had expression levels 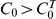 and *F* decreased. With small 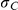 (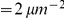 in Fig. 2), because the distribution of CCR5 expression was sharply peaked at 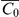, nearly all cells had 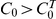 when 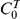 was modestly smaller than 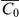, whereas few cells had 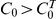 when 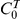 increased modestly above 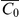. Consequently, *F* exhibited a sharp drop from 1 to 0 as 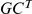 increased. The drop occurred around the value of 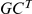 at which 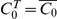. With larger 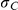, the wider distribution of CCR5 implied that the drop in *F* was gradual (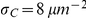 and 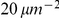 in Fig. 2). Eventually, as 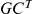 approached 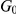, the expression level of gp120 on effector cells, 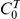 rose sharply (Fig. 2B (inset)) because of the limitation in the availability of gp120. Correspondingly, *F* dropped sharply as 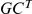 approached 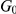 ( = 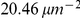 in Fig. 2B).

#### Gp120-CCR5 interactions in the presence of a coreceptor antagonist

**Figure 3:**
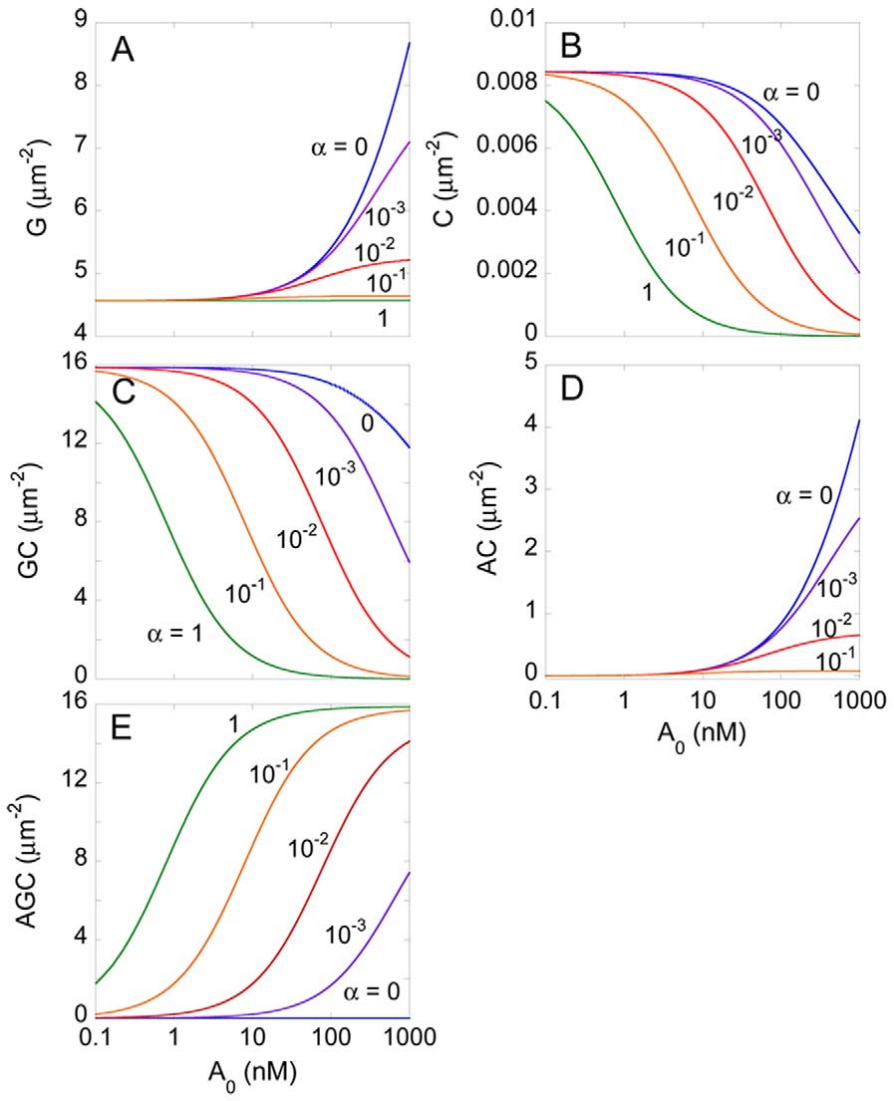
Predictions of the ternary complex model. Equilibrium surface densities of A) unbound gp120, *G*, B) unbound CCR5, *C*, C) gp120-CCR5 complexes, *GC*, D) CCR5-coreceptor antagonist complexes, *AC* and E) gp120-CCR5-coreceptor antagonist complexes, *AGC*, as functions of the concentration of the coreceptor antagonist, *A*_0_, for different values of the cooperativity factor, α, calculated by solving Eqs. (6)–(9).

We next predicted the equilibrium surface densities of the various reacting species across a single target cell-effector cell pair (Eq. 5), calculated using Eqs. (6)–(9), as functions of the concentration of the coreceptor antagonist, 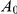, for different values of the cooperativity factor, 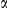 (Fig. 3). (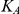 and 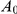 always appear as their product in the model equations, so that changes in 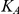 have the same effect as changes in 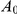.) 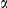 is the ratio of the binding affinities of the antagonist for gp120-bound CCR5 and unbound CCR5 (Eq. 5). For fixed 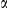, as we increased 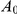, we found that the surface density of unbound gp120, 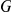, increased implying that fewer CCR5 molecules bound to gp120. At the same time, the surface densities of free CCR5 and gp120-CCR5 complexes, 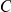 and 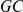, respectively, decreased, whereas the surface densities of their antagonist bound counterparts, 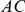 and 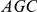, increased, indicating greater binding of the antagonist to CCR5. Higher values of 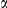 imply greater affinity of the antagonist for gp120-bound CCR5. Accordingly, for fixed 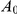, increasing 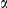 resulted in increased 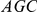, and decreased surface densities of all the other species. Thus, increasing 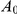 or decreasing 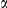 resulted in fewer CCR5 molecules binding gp120.

#### Cell-cell fusion in the presence of a coreceptor antagonist

**Figure 4:**
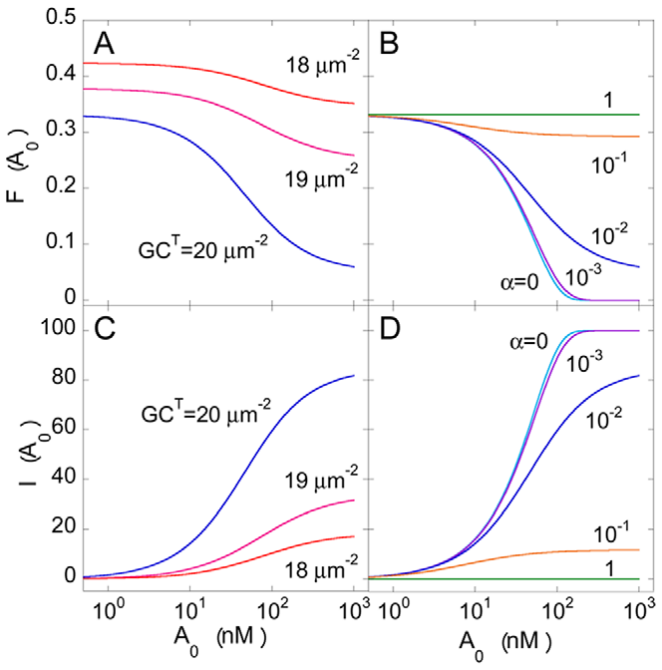
Model predictions of cell-cell fusion in the presence of a coreceptor antagonist. The fraction of cells fused, *F*(*A*_0_), as a function of the concentration of the coreceptor antagonist for A) different values of *GC^T^* with α = 0.01 and B) different values of α with *GC^T^* = 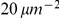 computed using Eqs (1)–(15). C) and D) The corresponding inhibition of fusion due to the coreceptor antagonist calculated using Eq. (16). The standard deviation of the CCR5 expression level, 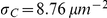.

In Fig. 4, we present the fraction of target cells fused, 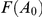, and the inhibition of fusion, 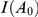, calculated using Eqs. (3)–(16), as functions of 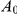, for different values of 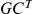 and 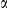. For fixed 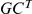 and 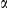, we found that, as expected, increasing 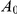 lowered 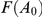 and increased 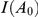. When 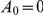, 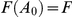, predicted above in the absence of the drug (Fig. 2B). Increasing 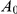 resulted in fewer gp120-CCR5 complexes, which lowered 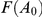 and increased 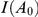. For very large values of 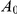, all CCR5 molecules were bound to the antagonist. Yet, because, according to the ternary complex model, the antagonist when bound to CCR5 lowers but does not annihilate the ability of CCR5 to bind gp120, gp120-CCR5 complexes formed even when all the CCR5 molecules were bound to the antagonist. Further, because we assumed that antagonist-bound CCR5 may also trigger fusion when bound to gp120, a fraction of cells, with sufficiently high expression levels of CCR5, fused even when 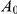 was very large. For given 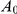 and 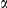, increasing 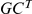 resulted in lower 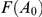 because the threshold expression level 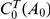 increased with 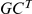 and fewer cells had expression levels 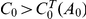. Accordingly, 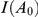 also increased with 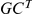. For fixed 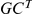 and 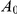, as 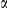 increased, greater binding of gp120 to antagonist-bound CCR5 increased 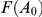 and consequently decreased 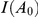.

Our model thus describes the outcome of a cell-cell fusion assay in the presence of a coreceptor antagonist. Below, we present comparisons of our predictions with experiments.

### Comparisons with experiments

#### Estimation of the threshold surface density of gp120-CCR5 complexes

**Figure 5:**
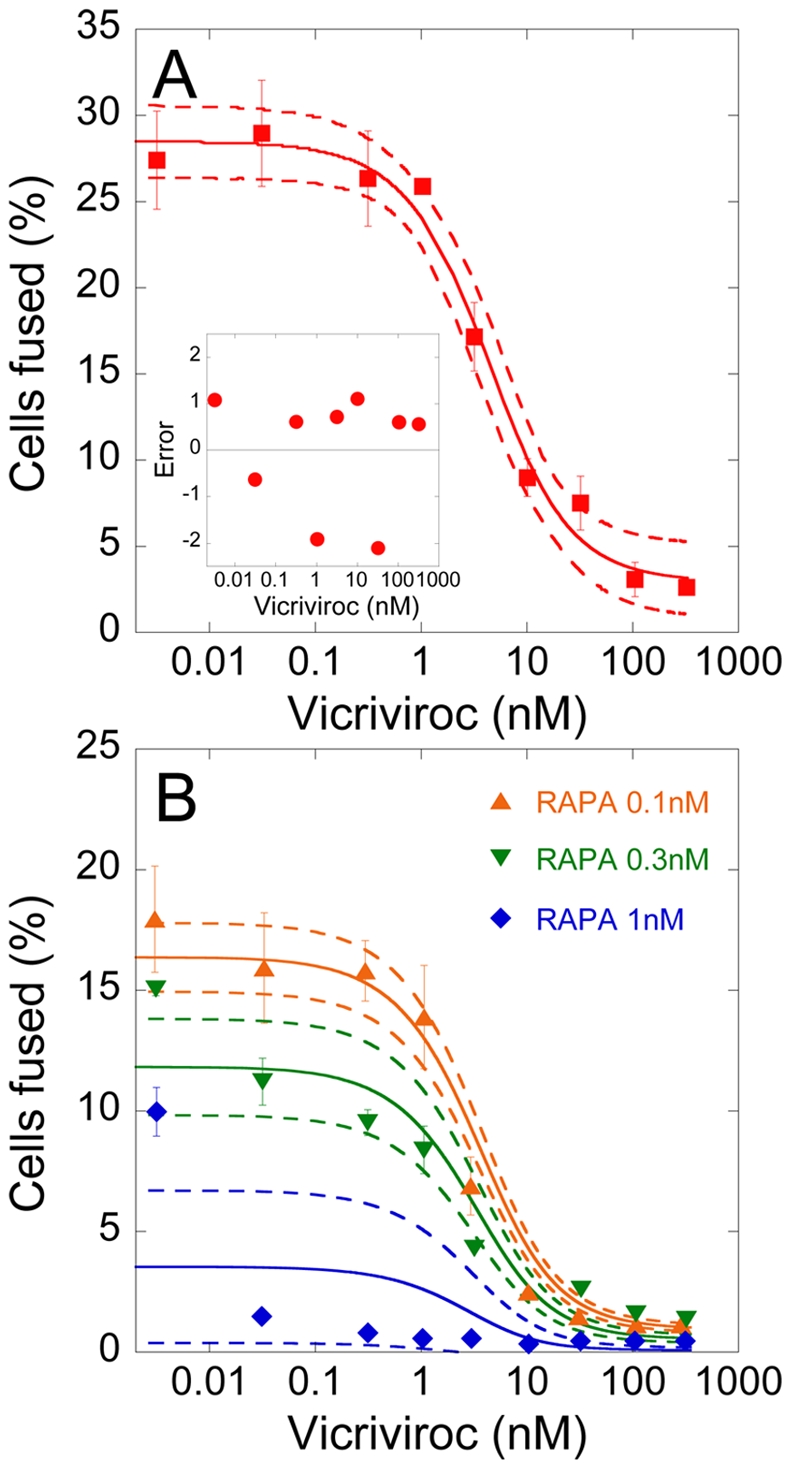
Comparisons of model predictions with experiments. A) Fit of model predictions of *F*(*A*_0_) (line) to published experimental data [24] (symbols) of the fraction of cells fused as a function of vicriviroc concentration using 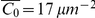 ( = 5318 molecules/cell [24]) and with 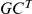, 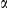, and 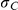 as adjustable parameters. The other parameters are mentioned in Methods. The dashed lines are 95% confidence limits on the predictions. Inset: Difference between model predictions and the experimental data; the mean error is 0.002 (in units of the percentage of cells fused) and is not significantly different from zero (*P* = 0.996 using a two-tailed t-test). B) Fits of model predictions of *F*(*A*_0_) (lines) to data [24] (symbols) of the fraction of cells fused as a function of vicriciroc concentration in the presence of different concentrations of rapamycin (RAPA) using 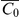 as an adjustable parameter. The other parameters are the same as in A). The best-fit parameter estimates are mentioned in the text.

Recently, Heredia *et al.* [24] performed cell-cell fusion assays to examine the antiviral activity of vicriviroc, a CCR5 antagonist in phase III trials (Gathe et al., Abstract # 45LB 17th Conference on Retroviruses and Opportunistic Infections, 2010). They performed the assays with and without rapamycin, a drug that lowers the expression level of CCR5 on cells [6] (Methods). We fit our prediction of 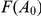 to their data of the percentage of cells fused as a function of vicriviroc concentration in the absence of rapamycin using three adjustable parameters, 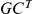, 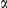, and 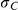. Our model provided excellent fits to the data (Fig. 5A). The best-fit parameter estimates (95% confidence intervals) were 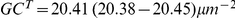, 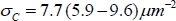, and 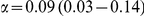. The best-fit estimate of 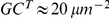 gives the minimum surface density of gp120-CCR5 complexes that must be formed between apposed cells for the cells to fuse. Further, using the best-fit value of 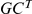 in Eq. (11), we obtained 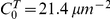, which yields the minimum expression level of CCR5 on target cells expressing excess CD4 that can fuse with effector cells with the gp120 expression level 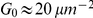. The latter minimum expression level corresponds to ∼6700 CCR5 molecules/cell (radius∼5 *µm*).

#### Validation of best-fit parameter estimates

To validate our parameter estimates, we compared our model predictions with independent data of cell-cell fusion as a function of vicriviroc concentration in the presence of rapamycin, reported by Heredia *et al.* [24]. Rapamycin lowers the mean expression level of CCR5 on target cells, 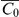, in a dose-dependent manner [6]. Using the above best-fit parameter estimates, we therefore fit model predictions to the data of Heredia *et al.* [24] using 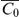 as an adjustable parameter. Our model provided good fits to the data (Fig. 5B) and yielded estimates of 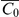 that are in close agreement with measurements, presenting a successful validation of our model and parameter estimates. Thus, for rapamycin levels of 0.1, 0.3, and 1 nM, we obtained 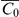 (95% CI) as 4300 (4100–4400), 3700 (3500–4000), and 2100 (1000–3200) molecules/cell, whereas experimental measurements of the mean CCR5 expression levels under the same conditions were 3900, 3535, and 2791, respectively [24].

#### Robustness of best-fit parameter estimates

**Figure 6:**
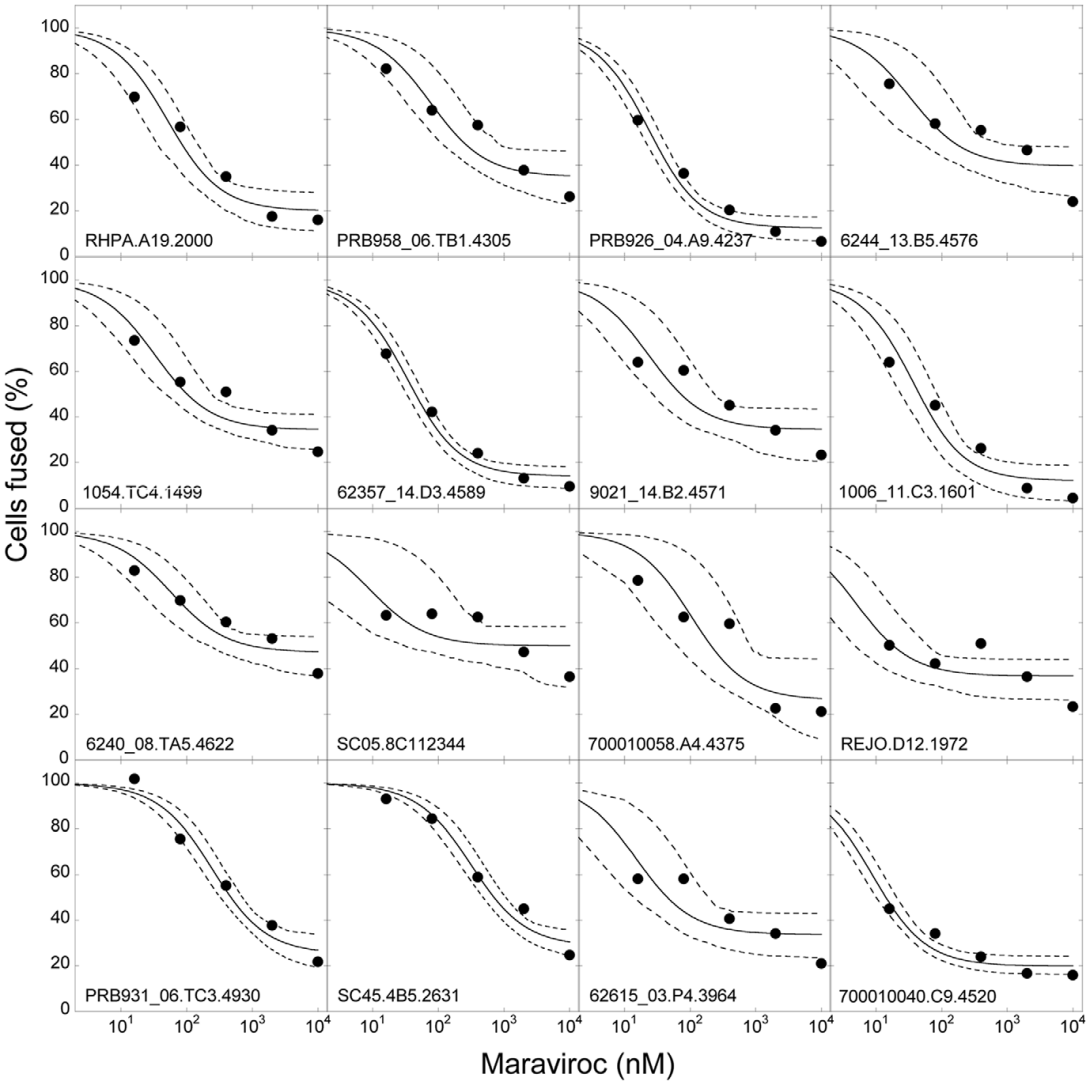
Robustness of model predictions. Fits of model predictions (lines) of the relative extent of cell-cell fusion, 100−*I*(*A*_0_), as a function of maraviroc concentration to published experimental observations [28] (symbols) using 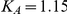 nM^−1^ and with 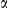 and 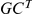 as adjustable parameters. The other parameters are mentioned in Methods. The different panels represent data from experiments using different Env clones (legends). The best fits (solid lines) and the corresponding 95% confidence limits (dashed lines) are shown. The best-fit parameter estimates are mentioned in Table 1.

The above experiments have employed the HIV-1 JRFL Env. Also, rapamycin is known to have a cytostatic effect on cells [6], the influence of which on cell-cell fusion remains unknown. To test the robustness of our parameter estimates, we therefore examined additional experiments on cell-cell fusion reported by Hu et al. [28] that employed a wide variety of Env clones derived from transmitted or are early founder viral genomes [29]. The experiments were performed in the presence of maraviroc, a coreceptor antagonist approved for clinical use, and the extent of cell-cell fusion relative to that in the absence of maraviroc was reported (Methods). We compared our predictions of the relative extent of cell-cell fusion, 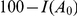, as a function of maraviroc concentration with the observations of Hu et al. [28] for 16 different Env clones. To describe this data, we employed the affinity of maraviroc for CCR5, *K_A_* = 1.15 nM^−1^ [30], and let the cooperativity factor, 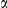, which is unknown for maraviroc, be an adjustable parameter. We also employed the threshold surface density of gp120-CCR5 complexes, 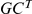, as an adjustable parameter to examine the dependence of this threshold on variations in the HIV-1 Env. Our model provided good fits to all the 16 data sets (Fig. 6). The best-fit values of 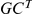 and 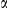 are presented in Table 1. We found that 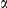 varied substantially across the different Env clones (range: 0.002–0.13) indicating varying degrees of sensitivity to maraviroc, in agreement with the conclusions of Hu et al. [28]. The varying sensitivity was also evident from the corresponding values of *IC_50_* (range: approximately 14–1300 nM), which we obtained for each clone as the maraviroc concentration at which the relative extent of fusion was 50% (Table 1). Interestingly, 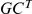 appeared to be nearly constant across the clones (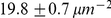) and close to the value estimated above (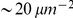) indicating the robustness of the latter estimate. That nearly the same value of 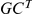 captured multiple experimental data sets with different HIV-1 Env clones in the presence of two different coreceptor antagonists and an agent that altered CCR5 expression levels suggests that our model captures the cell-cell fusion assays accurately and gives us confidence in our estimate of the threshold surface density of gp120-CCR5 complexes necessary for cell-cell fusion.

**Table 1:** Threshold surface density of gp120-CCR5 complexes for different Env clones.

Env clone	Threshold complex surface density, *GC^T^* (µm^−2^)	Cooperativity factor, *α*	IC_50_ (nM)
RHPA.A19.2000	19.9 (19.5–20.2)	0.011 (0.005–0.031)	79.5
PRB958_06.TB1.4305	19.6 (18.6–20.1)	0.009 (0.002–0.026)	242.1
PRB926_04.A9.4237	20.2 (20.1–20.3)	0.021 (0.012–0.033)	29.0
6244_13.B5.4576	20.0 (18.7–20.4)	0.020 (0.003–0.130)	143.3
1054.TC4.1499	20.1 (19.3–20.3)	0.020 (0.005–0.058)	100.9
62357_14.D3.4589	20.1 (20.0–20.2)	0.015 (0.009–0.021)	44.4
9021_14.B2.4571	20.2 (19.3–20.4)	0.028 (0.005–0.100)	71.4
1006_11.C3.1601	20.1 (19.7–20.3)	0.013 (0.005–0.030)	46.0
6240_08.TA5.4622	19.7 (18.5–20.2)	0.013 (0.004–0.040)	798.5
SC05.8C112344	20.3 (18.2–20.5)	0.082 (0.003–0.741)	1287.0
700010058.A4.4375	19.4 (16.4–20.2)	0.005 (0.001–0.030)	201.1
REJO.D12.1972	20.4 (20.2–20.5)	0.128 (0.032–0.473)	17.6
PRB931_06.TC3.4930	18.3 (17.4–19.0)	0.002 (0.001–0.004)	469.8
SC45.4B5.2631	17.8 (16.7–18.5)	0.002 (0.001–0.003)	699.3
62615_03.P4.3964	20.3 (19.5–20.4)	0.042 (0.007–0.200)	43.2
700010040.C9.4520	20.4 (20.3–20.4)	0.061 (0.038–0.096)	13.9
**Mean**	**19.8**	**0.030**	**267.9**

Estimates of the threshold surface density of gp120-CCR5 complexes, *GC^T^*, and the cooperativity factor, *α*, for 16 different transmitted or early founder viruses determined from fits of model predictions with experimental data [28] of cell-cell fusion in the presence of maraviroc (Fig. 6). 95% confidence intervals for the best-fit parameter estimates are shown in brackets. The corresponding *IC_50_* values of maraviroc are also listed.

## Discussion

The role of CCR5 in mediating HIV-1 entry has important implications for HIV-1 transmission and disease progression to AIDS as well as for strategies of intervention [1]–[3]. Yet, the threshold surface density of CCR5 molecules that must interact with gp120 to facilitate HIV-1 entry remains poorly estimated. Here, we constructed a mathematical model to analyse data from cell-cell fusion assays and estimated the threshold surface density of gp120-CCR5 complexes that enables HIV-1 Env-mediated cell-cell fusion. We found the threshold surface density of gp120-CCR5 complexes to be 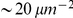. The corresponding minimum expression level of CCR5 on target cells that allows cell-cell fusion given the gp120 expression level on effector cells employed in our analysis and when CD4 is not limiting is 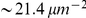, equivalent to ∼6700 molecules/cell (radius ∼5 *µm*). To validate our estimate, we analysed data from independent cell-cell fusion experiments performed in the presence of vicriviroc and maraviroc, both CCR5 antagonists, and rapamycin, a drug that down-regulated CCR5 expression, as well as with sixteen different Env clones derived from transmitted or early founder viruses. Our model provided good fits to the data and yielded an estimate of the threshold surface density of gp120-CCR5 complexes that remained nearly constant at 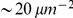 across these experiments, indicating the robustness of our estimate.

Our estimate of the threshold surface density of gp120-CCR5 complexes necessary for HIV-1 entry may facilitate optimal utilization of coreceptor antagonists for preventive and therapeutic intervention. For instance, the estimate suggests that a potent coreceptor antagonist must reduce the surface density of gp120-CCR5 complexes to below 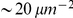. CCR5 expression levels vary substantially across individuals, with mean levels in the range ∼1000 to ∼10000 molecules/cell [31]. The variations may be due at least in part to variations in the CCL3L1 gene copy number, which was recently observed to be correlated with the susceptibility of individuals to HIV-1 infection [32]. Individuals with larger mean CCR5 expression levels would require greater drug exposure to achieve the same level of inhibition [33]. Our model may be applied to estimate the necessary drug exposure and may thus serve to personalize the usage of coreceptor antagonists based on the CCR5 expression level and/or the CCL3L1 gene dose in patients. Maraviroc was recently found to prevent transmission in rhesus macaques in a dose dependent manner when employed as a vaginal microbicide (Veazey et al., Abstract # 84LB 17th Conference on Retroviruses and Opportunistic Infections, 2010). CCR5 expression levels on target CD4^+^ T cells in mucosal regions may be higher than in peripheral blood [34]. Consequently, greater exposure to a coreceptor antagonist in mucosal regions may be necessary to prevent transmission than is necessary in plasma during treatment. Our model may again serve to quantify this greater exposure. Similarly, for vaccine strategies that aim to reduce the availability of target CD4^+^CCR5^+^ cells at sites of transmission [1], [2], our study suggests that CCR5 expression must be lowered to a level that restricts the formation of gp120-CCR5 complexes to below 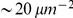 in order to prevent infection of target cells.

Our study may also inform the substantial ongoing efforts to elucidate the origins of the differences between SIV infection of natural and non-natural hosts (reviewed in [1]–[3]). An intriguing hypothesis explaining the non-progressive infection of natural hosts despite high plasma viral loads hinges on the reduced susceptibility of the central memory cell compartment in these animals due to low CCR5 expression [2]. In contrast, in non-natural hosts, the central memory compartment may be depleted more rapidly because of higher CCR5 expression levels. In both natural and non-natural hosts, the activated and effector memory cells have high CCR5 levels and are responsible for high plasma viral loads. The threshold expression level of CCR5 that renders target cells susceptible to SIV infection remains unknown. Our model may be applied to analyse data from SIV-Env-mediated cell-cell fusion assays and estimate the corresponding threshold CCR5 expression level for SIV infection, which may serve to elucidate the differences between natural and non-natural hosts of SIV. Indeed, more generally, our model provides a framework for analysing cell-cell fusion assays, widely employed to investigate HIV entry and related intervention strategies.

Recent studies have argued that the mechanism of viral entry into cells may be distinct from cell-cell fusion: while cell-cell fusion involves membrane fusion at the cell surface, HIV-1 entry appears to involve receptor and coreceptor mediated endocytosis [35], [36]. Following endocytosis, however, the viral membrane fuses with the endosomal membrane facilitating the release of viral contents into the cytoplasm leading to productive infection [35], [36]. Thus, if the membrane fusion processes are similar in cell-cell fusion and viral-endosomal fusion, which remains to be ascertained, the same threshold surface density of gp120-CCR5 complexes may underlie both cell-cell fusion and viral infection of target cells.

We recognize approximations in our model that hold for cell-cell fusion but may not apply to viral entry in vivo. First, our model describes the protein interactions that precede viral entry using a continuum, mass action-based approach. Such a continuum approximation is expected to be accurate for cell-cell fusion, where the number of protein molecules per cell is large (>10^3^). The advantage of the continuum approach is the simplicity of the resulting model equations and their facile application to data analysis. With virus-cell interaction, however, because virions express far fewer gp120 molecules (14±7 Env trimers per virion [37]), a stochastic description may be more appropriate (see, e.g., [38]). Second, our model considers the distribution of CCR5 expression levels across cells, but not of CD4 and gp120 levels. This approximation is reasonable for analysis of cell-cell fusion assays in the presence of coreceptor antagonists, which effectively lower the availability of CCR5 for binding gp120 and render CD4 and gp120 not limiting. With viral entry, however, a distribution of Env trimers is observed [37] and may have to be accounted for to accurately describe the susceptibility of target cells in vivo [20]. Further, where CD4 is down-modulated, as in African green monkeys [39], the assumption that all gp120 molecules are bound to CD4 and therefore accessible to CCR5 may not hold and the complete network of Env-CD4-CCR5 interactions (Text S1) may have to be considered. Indeed, cells with low CD4 expression have been suggested to require high CCR5 for infection [40]. Nonetheless, because the above approximations are expected to hold for the cell-cell fusion assays we analysed, they may not confound our estimate of the threshold surface density of gp120-CCR5 complexes necessary for viral entry.

The spatial distribution of CCR5 across the surface of a target cell and its role on viral entry remains to be established. While one study suggests that CCR5 molecules are localized within lipid rafts [41], another finds CCR5 molecules in non-raft regions [42]. Several studies suggest that CCR5 is colocalized and/or associated with CD4 [41], [43], [44]. Although CD4 is preferentially localized within rafts, such localization may not be essential for viral entry [45]. At the same time, membrane cholesterol depletion, which is known to affect raft formation and may also influence CCR5 mobility [46], inhibits viral entry when the receptors are not expressed in excess [41], [42], [47]. Current studies thus leave unclear the spatial distribution of CCR5 and its role in HIV-1 entry. Here, as an approximation, therefore, we have assumed CCR5 to be randomly distributed on the target cell surface. Further, effector cells have been suggested to recruit CCR5 to regions of cell-cell contact [41], the mechanisms underlying which remain unknown. Only recently have studies begun to unravel the local organization of protein complexes in the virus-cell contact region, which may be important for viral entry [38], [48], [49]. A quantitative assessment of the impact of the latter phenomena on estimates of the threshold surface density of gp120-CCR5 complexes necessary for viral entry awaits further studies that would establish the spatial distribution of CCR5 on target cells and of the mechanisms that underlie receptor migration and recruitment following virus-cell contact.

Finally, we note that our model assumed that each gp120 monomer in an Env trimer is independently accessible to CCR5. In contrast, steric constraints may result in increasingly hindered successive binding of CCR5 to the second and third gp120 monomers of an Env trimer. Conversely, cooperative binding may render successive binding easier [14]. Besides, recent single-molecule studies suggest that a complex energy landscape underlies gp120-CD4-CCR5 interactions [50], [51], the implications of which for viral entry remain to be fully understood. Further, heterogeneity in the CCR5 molecules, arising, for instance, due to post-translational modifications [52], may introduce additional variations in the affinity of CCR5 for gp120. Nonetheless, by assuming that all gp120 monomers are equally accessible to CCR5, our model ignores the association of gp120 into trimers and precludes identification of the stoichiometry of the Env-CD4-CCR5 complexes that renders the complexes fusion competent. At the threshold surface density, gp120-CCR5 complexes would be distributed such that some Env trimers are bound to 3 CCR5 molecules, some to 2, some to 1 and some to none (Fig. S1). It is possible that only Env trimers bound to 2 or more CCR5 molecules, for instance, mediate entry. While our model can be extended to predict this latter distribution (Text S1), currently available data does not allow establishment of the stoichiometry of CCR5 binding to Env that enables entry [20].

## Methods

### Data

We have analysed experimental data of HIV-1-Env mediated cell-cell fusion published recently by Heredia et al. [24] and Hu et al. [28]. In the cell-cell fusion assays reported by Heredia et al. [24], lymphocytes expressing CD4 and CCR5 (target cells) were treated with different concentrations of rapamycin and coincubated with 293T cells transfected with HIV-1 JRFL Env (effector cells) for 2.5 h at 37°C in the presence of known concentrations of vicriviroc. The two cell types were stained with different fluorescent dyes. Flow cytometry was used to detect cell-cell fusion: cells that were positive for both dyes indicated a fusion event. The fraction of target cells that eventually fused was reported as a function of vicriviroc concentration for different levels of exposure to rapamycin.

In the experiments performed by Hu et al. [28], QT6 cells transfected with CD4 and CCR5 (target cells) were exposed to QT6 cells transfected with HIV-1 Env expression constructs (effector cells) in the presence of different concentrations of maraviroc. The target cells were also transfected with a luciferase construct under the transcriptional control of T7 promoter. Luciferase activity was measured ∼8 h following co-incubation and reported as a percentage of the activity in the absence of maraviroc, thus representing the extent of inhibition of cell-cell fusion due to maraviroc. The experiments were performed using different Env clones that were derived from transmitted or early founder viral strains [29]. Hu et al. reported cell-cell fusion data for 18 different clones of which two were found to be highly resistant to maraviroc [28]. Here, we analysed data for the remaining 16 clones.

### Mathematical model

#### Single target cell-effector cell pair

We considered first the interactions between proteins across a single target cell-effector cell pair in the absence of a coreceptor antagonist (Fig. 1). Typically, CD4 molecules are in excess and rapidly bind all available gp120 molecules in the contact region on an apposed effector cell, as shown by our detailed reaction kinetics calculations (Text S1) and by independent simulations of virus-cell interactions [49]. We therefore considered the interactions between gp120 and CCR5:
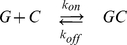
(1)Here, *G*, *C*, and *GC* are the surface densities (numbers per unit area) of gp120, CCR5, and gp120-CCR5 complexes, respectively. Eq. (1) assumes that each gp120 molecule in an Env trimer is bound to CD4 and is independently accessible to CCR5. The reaction in Eq. (1) attains equilibrium rapidly compared to fusion; equilibrium is attained within seconds (Fig. S1), whereas the lag time for fusion is in minutes [22]. At equilibrium, the surface densities of the reacting species obey
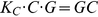
(2)where 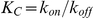 is the equilibrium association constant of CCR5 with gp120. If the effector cell expresses 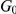 gp120 molecules per unit area (*i.e.*, 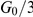 Env trimers per unit area) and the target cell 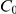 CCR5 molecules per unit area, then species balance implies
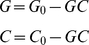
(3)where changes in the protein surface densities due to protein diffusion in and out of the cell-cell contact region are assumed negligible (see Discussion). Combining Eqs. (2) and (3) resulted in a quadratic equation in *GC*, solving which we obtained
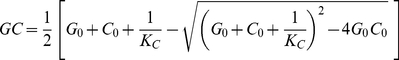
(4)Eq. (4) yields the surface density of CCR5 bound to gp120 between a single target cell-effector cell pair.

#### Threshold CCR5 binding for cell-cell fusion

We defined *GC^T^* as the minimum surface density of gp120-CCR5 complexes that must be formed between a target cell-effector cell pair for the cells to fuse. Thus, the cell pair fuses if *GC*≥*GC^T^*.

#### Single target cell-effector cell pair in the presence of a coreceptor antagonist

We next considered a single target cell-effector cell pair in the presence of a CCR5 antagonist, 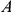, at concentration 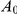. We employed the standard ternary complex model to describe the resulting allosteric interactions [27]:

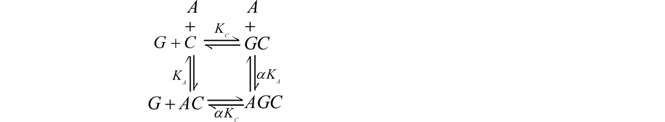
(5)


Here, gp120 can bind to a complex of CCR5 and *A*, denoted 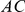, with an altered binding affinity 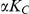, where 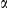 is the cooperativity factor. Similarly, 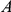 can bind to 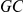 with affinity 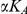, where 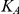 is the affinity of the antagonist for CCR5. At equilibrium, the ternary complex model yields
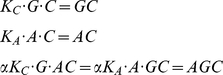
(6)along with the species balance equations
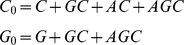
(7)


We assumed that the concentration of 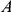 does not decrease substantially below 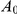. Combining Eqs. (6) and (7) yielded a quadratic equation in 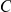, 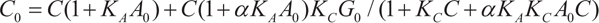, which we solved to obtain
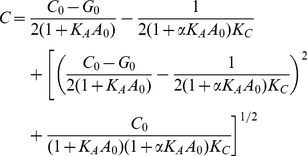
(8)Eqs. (6) and (7) also imply
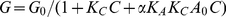
(9)using which in Eq. (6) along with Eq. (8), we obtained the surface densities of all the reacting species in the ternary complex model. In particular, the surface density of CCR5 bound to gp120,
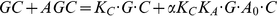
(10)For fusion, this latter surface density must be larger than the threshold surface density, 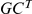.

#### Cell-cell fusion assay

In a cell-cell fusion assay, target cells with different expression levels of CCR5 form different surface densities of gp120-CCR5 complexes at equilibrium. We defined 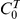 as that expression level of CCR5 that would result in the formation of the threshold surface density, 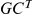, of complexes. In the absence of a coreceptor antagonist, from Eqs. (2) and (3), it follows that
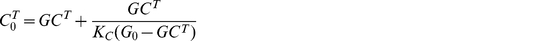
(11)Because 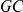 increases with 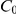 (Eq. (4)), all cells with 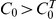 will fuse with effector cells. On the other hand, all cells with 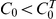 will be unable to fuse. We assumed next that the expression level of CCR5 on cells follows a truncated normal distribution with mean 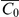 and standard deviation 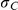,
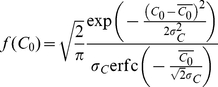
(12)where 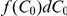 represents the fraction of cells with the CCR5 expression level within a small range 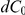 near 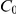, and 
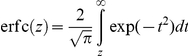
 is the complementary error function. Note that 
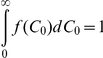
. The fraction of target cells that fuses, 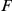, is then the fraction of cells with 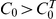, *i.e.*, 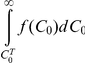, which upon substituting for 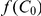 from Eq. (12) yielded
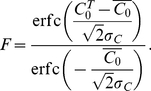
(13)Equation (13) predicts the fraction of cells fused in a cell-cell fusion assay in the absence of a coreceptor antagonist.

#### Cell-cell fusion assay in the presence of a coreceptor antagonist

In the presence of the coreceptor antagonist, the expression level of CCR5 that results in the formation of complexes at the surface density 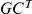 would be higher than 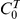 because the coreceptor antagonist inhibits the binding of CCR5 with gp120. We defined 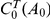 as that expression level of CCR5 that would result in the formation of the threshold surface density, 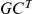, of complexes in the presence of the coreceptor antagonist at concentration 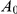. We obtained 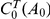 as that value of 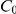 that satisfies
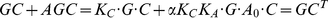
(14)The fraction of cells that fuses, 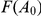, is then the fraction of cells with 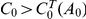, *i.e.*,
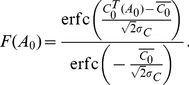
(15)Equation (15) predicts the fraction of target cells fused in a cell-cell fusion assay in the presence of a coreceptor antagonist. Note that Eq. (15) reduces to Eq. (13) when 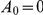. The percentage of inhibition of cell-cell fusion due to the coreceptor antagonist is then
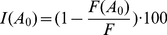
(16)


### Parameter estimates

We performed model calculations using parameter estimates representative of the cell-cell fusion assays we considered [24] unless mentioned otherwise. Target cells employed in the assays were lymphocytes from donors, with CCR5 expression levels in the range 2000–7000 molecules/cell [24]. We therefore assumed the mean CCR5 expression level on cells, 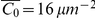, corresponding to ∼5000 molecules/target cell (radius∼5 *µm*). We set the expression level of gp120 on the effector cells, 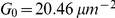, equivalent to ∼10000 Env trimers/effector cell (radius∼10 *µm*), following observations of ∼2 *ng* of gp120 on ∼10^6^ effector cells [22]. The equilibrium dissociation constant of gp120 binding to CCR5 is ∼4 nM [53]. The corresponding affinity when both gp120 and CCR5 are restricted to membranes, following the analysis of Bell [54] and assuming an encounter radius of 0.75 *nm*, is 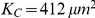 (Text S1). The affinity of vicriviroc for CCR5, 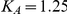 nM^−1^ [55]. The cooperativity factor, 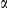, and the standard deviation of the CCR5 expression level on cells, 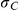, are not known and we estimated them along with the threshold surface density of complexes, 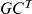, by fitting model predictions to data. The parameters employed are summarized in Table 2.

**Table 2:** Summary of model parameters and their values employed.

Parameter	Description	Value^a^	Source
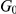	Surface density of gp120 on effector cells	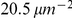	[22], see text
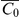	Mean surface density of CCR5 on target cells	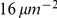	[24], see text
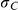	Standard deviation of the surface density of CCR5 across cells	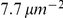	Best-fit (Fig. 5A)
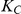	Equilibrium association constant of gp120 with CCR5	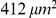	[53], see text
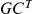	Threshold surface density of gp120-CCR5 complexes	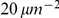	Best-fits (Figs. 5 and 6, Table 1)
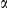	Cooperativity factor in the ternary complex model Eq.(5)	0.03	Best-fits (Fig. 6, Table 1)
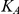	Equilibrium association constant of a coreceptor antagonist with CCR5	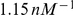	[30]

a Typical values; variations are indicated in the text and in figure legends.

### Model calculations and comparisons with experiments

We solved the above equations and fit model predictions to data using a computer program written in MATLAB. We employed the inbuilt routine NLINFIT, which uses the Levenberg-Margquardt algorithm for nonlinear least squares, for fitting model predictions to data and for obtaining 95% confidence intervals. For some of the data sets of transmitted/founder Env-mediated cell-cell fusion in the presence of maraviroc, NLINFIT yielded confidence intervals that included negative parameter values. We therefore determined 95% confidence intervals on the best-fit parameter values for the transmitted/founder Env-mediated cell-cell fusion data sets by performing 200 bootstrap replicates each, again in MATLAB.

## Supporting Information

Figure S1 **Time-evolution of the surface densities of the species in the reaction network.** Surface densities of the species in the network (Eq. (1) in Text S1), namely, (A) unbound Env and Env molecules bound to single CD4 molecules, (B) Env bound to 2 CD4 molecules, (C) Env bound to 3 CD4 molecules, and (D) unbound CD4 and CCR5, obtained by solving Eq. (2) in Text S1 (solid lines), and of the equilibrium surface densities of CCR5 and the gp120-CCR5 complexes in the simplified network (Eq. (5) in Text S1) (dashed lines), obtained by solving Eqs. (6)–(9) in Text S1. Parameter values and initial conditions are mentioned in Text S1. All surface densities are normalized with the initial Env surface density.
(TIF)

Text S1 **Detailed kinetics of Env-CD4-CCR5 binding.** A detailed description of the kinetics of the Env-CD4-CCR5 binding across a target cell-effector cell pair is presented.
(DOC)
