# Supplementary figures and images for: Estimating the Threshold Surface Density of Gp120-CCR5 Complexes Necessary for HIV-1 Envelope-Mediated Cell-Cell Fusion
Source: PLoS One. 2011 May 27;6(5):e19941. doi: 10.1371/journal.pone.0019941 (PMC3103592; doi:10.1371/journal.pone.0019941)

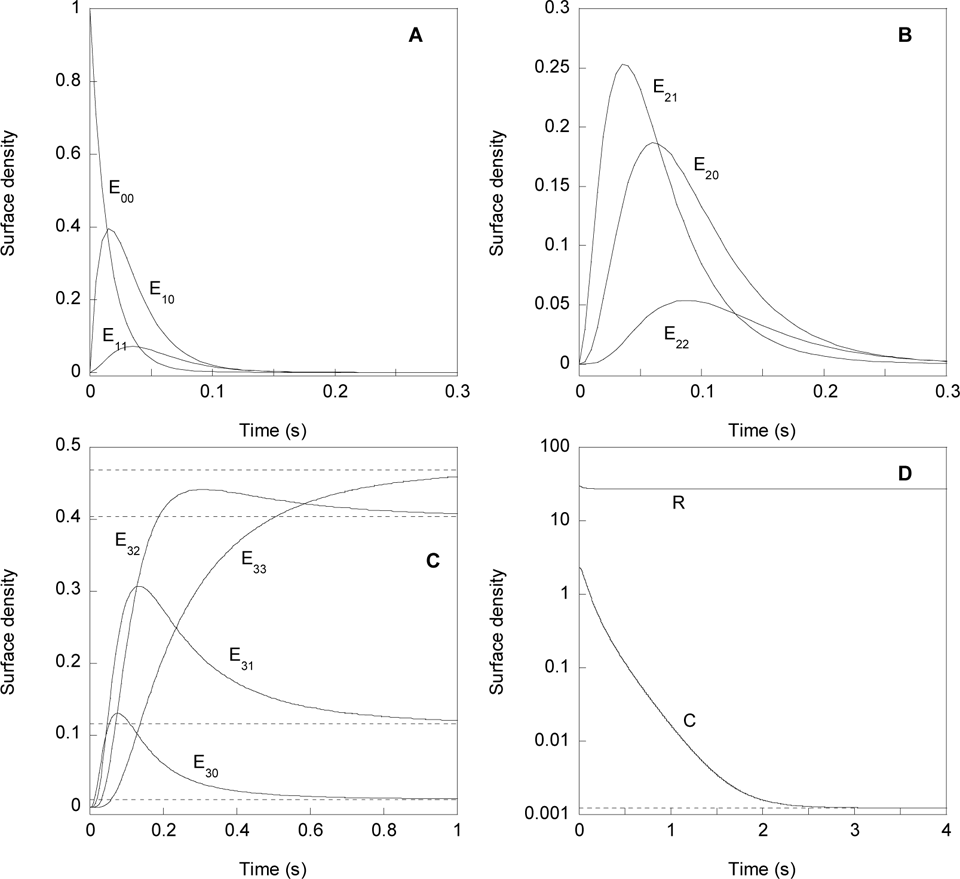

Supplement: Figure S1 — Time-evolution of the surface densities of the species in the reaction network. Surface densities of the species in the network (Eq. (1) in Text S1), namely, (A) unbound Env and Env molecules bound to single CD4 molecules, (B) Env bound to 2 CD4 molecules, (C) Env bound to 3 CD4 molecules, and (D) unbound CD4 and CCR5, obtained by solving Eq. (2) in Text S1 (solid lines), and of the equilibrium surface densities of CCR5 and the gp120-CCR5 complexes in the simplified network (Eq. (5) in Text S1) (dashed lines), obtained by solving Eqs. (6)–(9) in Text S1. Parameter values and initial conditions are mentioned in Text S1. All surface densities are normalized with the initial Env surface density. (TIF) [file pone.0019941.s001.tif]
