# Supplementary material for: Estimating the Threshold Surface Density of Gp120-CCR5 Complexes Necessary for HIV-1 Envelope-Mediated Cell-Cell Fusion
Source: PLoS One. 2011 May 27;6(5):e19941. doi: 10.1371/journal.pone.0019941 (PMC3103592; doi:10.1371/journal.pone.0019941)
Supplement: Text S1 — Detailed kinetics of Env-CD4-CCR5 binding. A detailed description of the kinetics of the Env-CD4-CCR5 binding across a target cell-effector cell pair is presented. (DOC) [file pone.0019941.s002.doc]

**Supporting Information**

**Text S1. Detailed kinetics of Env-CD4-CCR5 binding**

***Reaction network***

Here, we describe the reaction kinetics of the binding of Env on an effector cell to CD4 and CCR5 on a closely apposed target cell. Let the effector cell have *E*0 Env trimers and the target cell *R*0 CD4 and *C*0 CCR5 molecules, respectively, per unit area. Let *Eij* be the surface density (number per unit area) of Env trimers that are bound to *i* CD4 and *j* CCR5 molecules, respectively, where $0\leq j\leq i\leq3$. Thus, for instance, *E*00 are unbound Env trimers and *E*33 are trimers bound to the maximum of 3 CD4 and 3 CCR5 molecules. We recognize that CCR5 molecules can bind to gp120 only after CD4 binding so that $j\leq i$. The reactions between Env, CD4 and CCR5 are summarized in the network below.


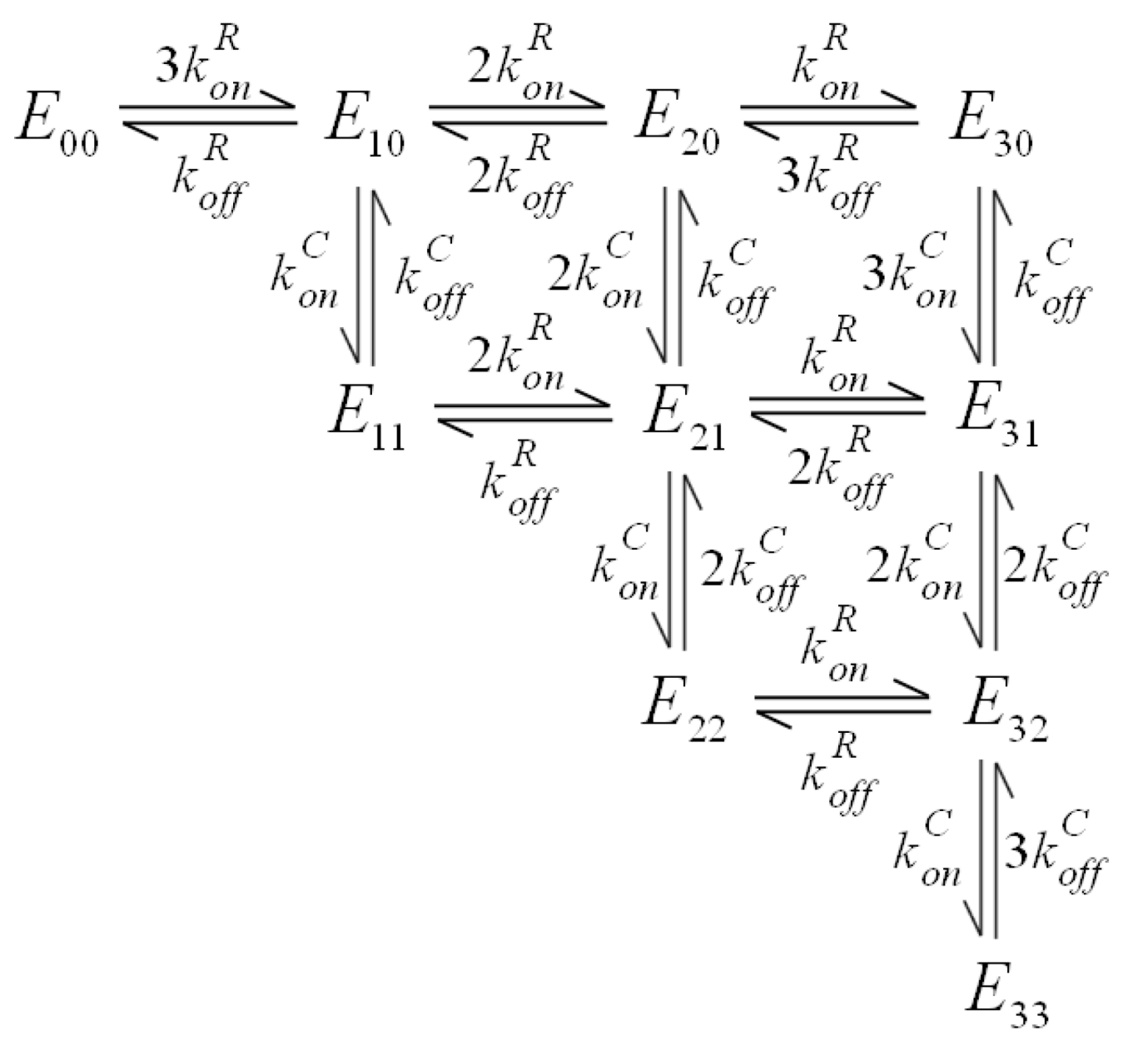


Here, the horizontal arms represent CD4 binding and unbinding, and vertical arms represent CCR5 binding and unbinding, respectively. CD4 and CCR5 are not shown for convenience. The time evolution of the surface density of each of the species in the reaction network was obtained by solving the following rate equations

$$\begin{matrix} \frac{dE_{00}}{dt}=-3k_{on}^{R}E_{00}R+k_{off}^{R}E_{10} \\ \frac{dE_{10}}{dt}=3k_{on}^{R}E_{00}R-k_{off}^{R}E_{10}-2k_{on}^{R}E_{10}R+2k_{off}^{R}E_{20}-k_{on}^{C}E_{10}C+k_{off}^{C}E_{11} \\ \frac{dE_{11}}{dt}=-2k_{on}^{R}E_{11}R+k_{off}^{R}E_{21}+k_{on}^{C}E_{10}C-k_{off}^{C}E_{11} \\ \frac{dE_{20}}{dt}=2k_{on}^{R}E_{10}R-2k_{off}^{R}E_{20}-k_{on}^{R}E_{20}R+3k_{off}^{R}E_{30}-2k_{on}^{C}E_{20}C+k_{off}^{C}E_{21} \\ \frac{dE_{21}}{dt}=2k_{on}^{R}E_{11}R-k_{off}^{R}E_{21}-k_{on}^{R}E_{21}R+2k_{off}^{R}E_{31}+2k_{on}^{C}E_{20}C-k_{off}^{C}E_{21}-k_{on}^{C}E_{21}C+2k_{off}^{C}E_{22} \\ \frac{dE_{22}}{dt}=-k_{on}^{R}E_{22}R+k_{off}^{R}E_{32}+k_{on}^{C}E_{21}C-2k_{off}^{C}E_{22} \\ \frac{dE_{30}}{dt}=k_{on}^{R}E_{20}R-3k_{off}^{R}E_{30}-3k_{on}^{C}E_{30}C+k_{off}^{C}E_{31} \\ \frac{dE_{31}}{dt}=k_{on}^{R}E_{21}R-2k_{off}^{R}E_{31}+3k_{on}^{C}E_{30}C-k_{off}^{C}E_{31}-2k_{on}^{C}E_{31}C+2k_{off}^{C}E_{32} \\ \frac{dE_{32}}{dt}=k_{on}^{R}E_{22}R-k_{off}^{R}E_{32}+2k_{on}^{C}E_{31}C-2k_{off}^{C}E_{32}-k_{on}^{C}E_{32}C+3k_{off}^{C}E_{33} \\ \frac{dE_{33}}{dt}=k_{on}^{C}E_{32}C-3k_{off}^{C}E_{33} \\ \frac{dR}{dt}=-3k_{on}^{R}E_{00}R+k_{off}^{R}E_{10}-2k_{on}^{R}E_{10}R+2k_{off}^{R}E_{20}-k_{on}^{R}E_{20}R+3k_{off}^{R}E_{30}-2k_{on}^{R}E_{11}R+k_{off}^{R}E_{21} \\ -k_{on}^{R}E_{21}R+2k_{off}^{R}E_{31}-k_{on}^{R}E_{22}R+k_{off}^{R}E_{32} \\ \frac{dC}{dt}=-k_{on}^{C}E_{10}C+k_{off}^{C}E_{11}-2k_{on}^{C}E_{20}C+k_{off}^{C}E_{21}-k_{on}^{C}E_{21}C+2k_{off}^{C}E_{22}-3k_{on}^{C}E_{30}C+k_{off}^{C}E_{31} \\ -2k_{on}^{C}E_{31}C+2k_{off}^{C}E_{32}-k_{on}^{C}E_{32}C+3k_{off}^{C}E_{33} \end{matrix}$$

Here, *R* and *C* are the surface densities of unbound CD4 and CCR5 molecules, respectively. $k_{on}^{R}$ is the association rate constant of CD4 and monomeric gp120 and $k_{off}^{R}$ is the dissociation rate constant of the gp120-CD4 complex. Similarly, $k_{on}^{C}$ is the association rate constant of CCR5 with a gp120-CD4 complex and $k_{off}^{C}$ is the dissociation rate constant of CCR5 from the gp120-CD4-CCR5 complex. We assumed that each of the gp120 monomeric units in an Env trimer interacts independently with CD4 and subsequently with CCR5. Thus, the rate constant for the conversion of *E*30 to *E*20, for instance, is $3k_{off}^{R}$ because each of the 3 CD4 molecules bound to gp120 in *E*30 dissociates with the rate constant $k_{off}^{R}$. Further, we assumed that CD4 from a gp120-CD4-CCR5 complex dissociates after the dissociation of CCR5, which ensures $j\leq i$. The forward and backward rate constants of each of the reactions in the network are thus determined in terms of $k_{on}^{R}$, $k_{off}^{R}$, $k_{on}^{C}$ and $k_{off}^{C}$, which we estimated as follows.

***Determination of rate constants***

We employed the analysis of Bell [1] to determine the reaction rate constants $k_{on}^{R}$, $k_{off}^{R}$, $k_{on}^{C}$ and $k_{off}^{C}$ when the proteins are restricted to apposed membranes, given the corresponding rate constants when the proteins are freely diffusing in solution. Let *ron­* and *roff* be the association rate constant of gp120 and CD4 and the dissociation rate constant of the gp120-CD4 complex, respectively, when gp120 and CD4 are in solution. Then, from Bell’s analysis, it follows that

$k_{on}^{R}=\frac{d_{+}^{m}}{d_{+}^{s}}\frac{\left(d_{-}^{s}+r_{+}\right)}{\left(d_{-}^{m}+r_{+}\right)}r_{on}$ and $k_{off}^{R}=\frac{d_{-}^{m}}{d_{-}^{s}}\frac{\left(d_{-}^{s}+r_{+}\right)}{\left(d_{-}^{m}+r_{+}\right)}r_{off}$

where $r_{+}=\frac{r_{on}d_{-}^{s}}{d_{+}^{s}-r_{on}},$ $r_{-}=\frac{r_{off}d_{+}^{s}}{d_{+}^{s}-r_{on}},$ $d_{+}^{s}=4\pi\left[D_{s}(E)+D_{s}(R)\right]R_{ER},$ $d_{-}^{s}=3\left[D_{s}(E)+D_{s}(R)\right]R_{ER}^{-2},$ $d_{+}^{m}=2\pi\left[D_{m}(E)+D_{m}(R)\right],$ and $d_{-}^{m}=2\left[D_{m}(E)+D_{m}(R)\right]R_{ER}^{-2}$. Here, *Ds*(*R*) and *Dm*(*R*) are the diffusivities of CD4molecules in solution and in the membrane, respectively, *Ds*(*E*) and *Dm*(*E*) are the corresponding values for Env, and *RER* is the encounter distance, the minimum proximity between Env and CD4 for bond formation.

Similarly, if *con­* and *coff* are the association rate constant of CCR5 with the gp120-CD4 complex and the dissociation rate constant of CCR5 from the gp120-CD4-CCR5 complex, respectively, when gp120-CD4 and CCR5 are in solution, then

$k_{on}^{C}=\frac{d_{+}^{m}}{d_{+}^{s}}\frac{\left(d_{-}^{s}+c_{+}\right)}{\left(d_{-}^{m}+c_{+}\right)}c_{on}$ and $k_{off}^{C}=\frac{d_{-}^{m}}{d_{-}^{s}}\frac{\left(d_{-}^{s}+c_{+}\right)}{\left(d_{-}^{m}+c_{+}\right)}c_{off}$

where $c_{+}=\frac{c_{on}d_{-}^{s}}{d_{+}^{s}-c_{on}},$ $c_{-}=\frac{c_{off}d_{+}^{s}}{d_{+}^{s}-c_{on}},$ $d_{+}^{s}=4\pi\left[D_{s}(ER)+D_{s}(C)\right]R_{ERC},$ $d_{-}^{s}=3\left[D_{s}(ER)+D_{s}(C)\right]R_{ERC}^{-2},$ $d_{+}^{m}=2\pi\left[D_{m}(ER)+D_{m}(C)\right],$ and $d_{-}^{m}=2\left[D_{m}(ER)+D_{m}(C)\right]R_{ERC}^{-2}$. Again, *Ds*(*C*) and *Dm*(*C*) are the diffusivities of CCR5molecules in solution and in the membrane, respectively, *Ds*(*ER*) and *Dm*(*ER*) are the corresponding values for the gp120-CD4 complex, and *RERC* is the encounter distance, the minimum proximity between gp120-CD4 and CCR5 for bond formation.

The rate constants in solution have been measured. Thus, *ron­*=6.72×10^4^ M-1s-1 and *roff*=1.5×10-3 s-1 [2]. Further, *coff/con*= 4 nM (*K*d) and *t1/2*=*ln*(2)/*coff* = 5.8 min [3], which yield *con*=5×10^5^ M-1s-1 and *coff*=2×10-3 s-1. The diffusivity of CD4 in solution, following the Stokes-Einstein equation, is *Ds*(*R*)=85 µm2s-1 [4]. We assumed the diffusivities of all the other proteins and complexes in solution to be equal to *Ds*(*R*). The diffusivities of CD4 and CCR5 as well as the gp120-CD4 complex on membranes have been measured; *Dm*(*R*)=52 µm2s-1 and *Dm*(*C*)= 45 µm2s-1 [5], and *Dm*(*ER*)= 0.05 µm2s-1 [6]. As an approximation, we set *Dm*(*E*)=*Dm*(*R*). With these parameter values and with *RER=RERC*=0.75 nm [1], we obtained $k_{on}^{R}$=0.11 µm2s-1, $k_{off}^{R}$=1.5×10-3 s-1, $k_{on}^{C}$=0.83 µm2s-1 and $k_{off}^{C}$=2×10-3 s-1.

***Kinetics of the Env-CD4-CCR5 interactions***

We solved the above equations (Eq. 2) with the following initial conditions: *E*00(0)=*E*0=6.82 µm-2 (=*G*0*/*3, see manuscript), *Eij*(0)=0 for all *i*, *j*>0, *R*(0)=*R*0=207 µm-2 (corresponding to 65000 CD4 molecules/cell [7]), and *C*(0)=*C*0=16 µm-2 (corresponding to ~5000 CCR5 molecules/cell, see manuscript). We solved the equations using a computer program written in C. The resulting time evolution of each of the proteins and complexes is presented in Fig. S1.

After the initiation of the reactions, *E*00, the surface density of unbound Env, dropped sharply (in ~0.05 s) and *E*10 and *E*11 rose, indicating binding of CD4 and CCR5 to Env (Fig. S1A). By ~0.1 s, *E*10 and *E*11 also dropped and made way for *E*20,*E*21, and *E*22, indicating continued CD4 and CCR5 binding (Figs. S1A and S1B). By ~0.3s, *E*20,*E*21, and *E*22 declined, with only *E*30,*E*31, *E*32 and ­*E*33 remaining in significant numbers (Figs. S1B and S1C). Thus, the reactions reached equilibrium in ~1 s (Fig. S1C), and at equilibrium all Env trimers were bound to 3 CD4 molecules. That CD4 was in large excess is indicated by the negligible drop in its surface density (Fig. S1D). CCR5 molecules, however, were not in excess (Fig. S1D); consequently, a distribution of Env trimers bound to 3, 2, 1, and no CCR5 molecules emerged. We derived analytical expressions for the surface densities of the complexes at equilibrium, which we present below.

***Reaction equilibrium with excess CD4***

With excess CD4, all gp120 molecules were bound to CD4 so that only CCR5 binding and unbinding to Env need be considered. The reaction network (Eq. (1)) then simplified to

At equilibrium, we therefore have

$$\begin{matrix} E_{31}=3K_{C}E_{30}C \\ E_{32}=K_{C}E_{31}C \\ E_{33}=(K_{C}/3)E_{32}C \end{matrix}$$

where *KC*=$k_{on}^{C}$/$k_{off}^{C}$. In addition, species balance implies

$$\begin{matrix} E_{0}=E_{30}+E_{31}+E_{32}+E_{33} \\ C_{0}=C+E_{31}+2E_{32}+3E_{33} \end{matrix}$$

Combining Eqs. (6) and (7) resulted in a quadratic equation in *C*, which we solved to obtain

$$C=\frac{-(K_{C}(3E_{0}-C_{0})+1)+\sqrt{(K_{C}(3E_{0}-C_{0})+1)^{2}+4K_{C}C_{0}}}{2K_{C}}$$

Eqs. (6) and (7) also imply

$$E_{30}=\frac{E_{0}}{(1+K_{C}C)^{3}}$$

Substituting Eqs. (8) and (9) in Eq. (6) then yielded the equilibrium surface densities of each of the complexes in Eq. (5). The resulting predictions (dashed lines in Fig. S1C and D) are in agreement with the equilibrium surface densities predicted by the dynamical equations (Eq. (2)) above.

In summary, reaction equilibrium was attained rapidly (~1 s) compared to the time required for cell-cell fusion (~min). Further, all gp120 molecules were bound to CD4 at equilibrium. It follows that if each gp120 molecule were to interact independently with CCR5, then the reaction scheme in Eq. (5) may be simplified further by ignoring the trimeric nature of Env and considering the total surface density of gp120 molecules as available for interaction with CCR5. The latter simplification is employed in the manuscript (Eq. (1)).

**Supplementary References**

1. Bell GI (1978) Models for the specific adhesion of cells to cells. Science 200: 618-627.

2. Myszka DG, Sweet RW, Hensley P, Brigham-Burke M, Kwong PD, et al. (2000) Energetics of the HIV gp120-CD4 binding reaction. Proc Natl Acad Sci U S A 97: 9026-9031.

3. Doranz BJ, Baik SSW, Doms RW (1999) Use of a gp120 binding assay to dissect the requirements and kinetics of human immunodeficiency virus fusion events. J Virol 73: 10346.

4. English TJ, Hammert DA (2004) Brownian adhesive dynamics (BRAD) for simulating the receptor-mediated binding of viruses. Biophys J 86: 3359-3372.

5. Baker AM, Sauliere A, Gaibelet G, Lagane B, Mazeres S, et al. (2007) CD4 interacts constitutively with multiple CCR5 at the plasma membrane of living cells: A fluorescence recovery after photobleaching at variable radii approach. J Biol Chem 282: 35163-35168.

6. Pal R, Nair BC, Hoke GM, Sarngadharan MG, Edidin M (1991) Lateral diffusion of CD4 on the surface of a human neoplastic T-cell line probed with a fluorescent derivative of the envelope glycoprotein (gp120) of human immunodeficiency virus type 1 (HIV-1). J Cell Phys 147: 326-332.

7. Doms RW (2000) Beyond receptor expression: the influence of receptor conformation, density, and affinity in HIV-1 infection. Virology 276: 229-237.
